# Development of A Caregiver‐Reported Scale for Pediatric Cancer Financial Toxicity (CRS‐PCFT)

**DOI:** 10.1002/cam4.70675

**Published:** 2025-02-13

**Authors:** Pengfei Li, Nan Zhang, Xinyue Xu, Yan Liu, Zhengyang Lu, Qian Gao, Shihong Lin, Weimin Guan, Wenxuan Yan, Boyu Liu, Youhua Lu, Jinming Yu

**Affiliations:** ^1^ School of Public Health Shandong Second Medical University Weifang China; ^2^ Cancer Hospital of Shandong First Medical University (Shandong Cancer Institute, Shandong Cancer Hospital) Jinan China; ^3^ School of Public Health and Health Administration, Shandong First Medical University & Shandong Academy of Medical Science Jinan China

**Keywords:** financial toxicity, pediatric cancer, scale development

## Abstract

**Background:**

Financial toxicity is common among families of pediatric patients with cancer. However, the availability of survey and/or screening instruments specific to pediatric family financial toxicity is limited.

**Methods:**

A two‐round cross‐sectional survey was conducted in Shandong Province, China. We combined classical test theory (CTT) and item response theory (IRT) to validate items of the caregiver‐reported scale for pediatric cancer financial toxicity (CRS‐PCFT) after Delphi. The scale structure, reliability, and validity were determined and validated by exploratory factor analysis (EFA) and confirmatory factor analysis (CFA). The threshold was discussed based on the correlations between CRS‐PCFT and socio‐factors.

**Findings:**

A 16‐item initial scale was determined after Delphi. The data from 206 pilot survey samples was used to select and validate items, and a 10‐item CRS‐PCFT was developed. The scale showed satisfactory reliability and validity based on data from 398 formal survey samples. When the CRS‐PCFT scores were into high and low toxicity groups by the median, they were significantly correlated with education (*r* = −0.284, *p* < 0.0001), household income (*r* = −0.253, *p* < 0.0001), work status (*r* = −0.173, *p* = 0.001), and cancer stages (*r* = 0.147, *p* = 0.003).

**Interpretation:**

CRS‐PCFT demonstrates robust reliability and validity and makes it more accurate to obtain the pediatric cancer financial toxicity conditions. Additional research should be done to validate CRS‐PCFT.

## 1 Introduction

Pediatric cancer financial toxicity (PCFT) is a serious challenge faced by related families and society [1, 2, 3]. The financial hardship, psychological response, and coping behaviors are the main aspects of financial toxicity [4]. The cost of pediatric cancer treatment can lead to severe financial distress for this population [5, 6, 7]. In 2018, the World Health Organization launched the global initiative for childhood cancer (GICC), highlighting the reduction of the economic impact on families affected by childhood cancer as a key focus [8]. However, public attention to financial toxicity predominantly focuses on the adult population, rather than the patients with pediatric cancer [2, 9, 10].

Compared to the adult population, pediatric cancer financial toxicity is significantly more severe [8]. A Canadian research study reveals that when a child is diagnosed with cancer, the economic impact has long‐term effects on the financial security, quality of life, and future well‐being of the entire family [11]. Accurately measuring pediatric cancer financial toxicity is fundamental and essential for conducting relevant research. To date, the comprehensive score for financial toxicity (COST) is the most widely used instrument for measuring pediatric cancer financial toxicity [12, 13, 14]. However, the COST scale was developed based on patient‐reported outcomes, and its item descriptions are not suitable for the specific circumstances of children with cancer and caregivers [12]. As of now, there is no specific instrument designed to measure pediatric cancer financial toxicity.

## 2 Methods

### 2.1 Item Source of CRS‐PCFT

Item generation defines the instrument's contents and quality and ensures all important items are included [12, 15]. We have conducted a thorough literature search to gather all widely recognized financial toxicity scales and measurement instruments. Based on the literature search results, we completed an interview outline containing 8 topics. A semi‐structured qualitative interview of 20 caregivers (15 mothers and 5 fathers) was conducted in April 2024. 62 initial scale items were collected from established financial toxicity scales and caregivers interview materials. Scales included the comprehensive scores for financial toxicity based on the patient‐reported outcome measures (COST‐PROM, 11 items) [12, 16], incharge financial distress/financial well‐being scale (IFDFW, 10 items) [17], personal financial wellness scale (PFW, 8 items) [18], Financial Index of Toxicity (FIT, 9 items) [19], breast cancer finances survey (BCFS, 42 items) [20], and subjective financial distress questionnaire (SFDQ, 14 items) [21]. 3 items (a., if necessary, consideration will be given to covering treatment expenses by selling assets such as houses and cars. b., my family members may go to re‐work or get a part‐time job to relieve the pressure of medical expenses. c., I feel anxious about the potential decrease in income or job loss for myself or my family members due to childcare responsibilities) were extracted from the interview data and included as supplements during item generation.

### 2.2 Delphi Techniques

A two‐round Delphi consultation was conducted to finalize and validate the items. 23 experts come from the clinical, nursing, public health, and management fields. In the first round of consultation, experts received consultation emails that included all initial items. We adjusted (merge, remove, and add) items based on experts' opinions and emailed back the revised results. In the second round, the experts' modification opinions reached a consensus, and we stopped it. The criteria of assessment (Ca) scored 0.883, familiarity degree (Cs) scored 0.783, and the contrast ratio (Cr) scored 0.833.

### 2.3 Item Importance Evaluation

Fifty‐nine caregivers were invited to complete the item importance evaluation. Each item corresponds to 0 (unimportant) and 1 (important) options. Participants evaluate whether each item is important or not. Item importance rates were arranged, and that item will be removed if the importance rate is less than 80%.

### 2.4 Sample Size and Participants

Sixteen items were retained as the main content of the initial scale after Delphi and importance evaluation. The pilot survey sample size should be 5–10 times the number of scale items in the pilot survey. The sample size of scale validation needs to meet the requirements of the cross‐sectional survey shown in Equation (1).

(1) $$n=\frac{Z_{1-\alpha /2}^{2}P\left(1-P\right)}{d^{2}}$$

According to the normal distribution, we defined *α* = 0.05, *Z* = 1.96. *P* reflects the incidence of financial toxicity. Research based on Chinese data reveals the prevalence of financial toxicity in the Chinese population is 80% or more [22, 23]. *d* represents the allowable error of sample extraction, and we defined the value of *d* as 0.05*P. According to Equation (1), the scale validation should include a sample size of at least 384 or more.

Caregivers of the patients with pediatric cancer aged 0–14 were invited as participants, and they were sourced from two comprehensive hospitals and one specialized tumor hospital in Shandong Province, China. A child who has not received a pathological diagnosis or anti‐tumor treatment was ruled out. To ensure the accuracy of data, investigators received professional and standard training before the investigation. Nurses determined the investigation time for each caregiver to minimize the influence of relaxation or treatment as much as possible. The questionnaire was completed through self‐completion. It was designed as a question‐and‐answer session for caregivers who can not read. The pilot survey was conducted in July 2024 and 206 caregivers effectively finished the questionnaire. After that, 398 caregivers completed the formal survey questionnaire in August and September 2024. We required the participants' sociodemographic characteristics and the degree of initial items. All participants had signed the consent form. The study protocol was provided by the local Institutional Review Boards (No. SDTHEC2023009018).

### 2.5 Sociodemographic Characteristics

Participants provided main sociodemographic data (including sex, age, and native place of children. Sex, age, marital status, education, career, and work status of caregivers, etc.). We also asked for information about their family's economic income.

### 2.6 Item Analysis

The data for item validation analyses comes from 206 caregivers in the pilot survey. Item values were collected on a 5‐point Likert scale from 0 (not at all) to 4 (absolutely consistent) with a 2‐week time window. Positive items were scored in reverse to maintain consistency in the scoring direction. The higher the item score, the more severe the financial toxicity.

A quantitative item analysis was completed to determine the scale's reliability and validity. The structure validity analysis methods include critical ratio (CR), interitem correlation (IIC), and item‐total correlation (ITC). The internal consistency was confirmed using reliability analysis indexes such as corrected item‐total correlation (CITC) and Cronbach α. The observation indexes fitted by the graded response model (GRM) included discrimination parameters (a), difficulty parameters (b), and item information amount.

The top 27% of the scale's total score and the bottom 27% are divided into two groups. CR requires a significant correlation between the scores of each item in two groups in an independent‐sample *t*‐test (*p* < 0.05). IIC and ITC are necessary statistical methods for testing correlations between items, and items with total scores. If the IIC of a pair of items is greater than 0.7, the one with a lower importance score should be considered for exclusion. The ITC needs to be greater than 0.3 and significant. Cronbach α is the primary observational metric for reliability analysis. If the scale's overall Cronbach α increases after an item is deleted, the item should be considered for deletion. Furthermore, each item's CITC must also be higher than 0.4. The GRM is the most common model for IRT to cope with multilevel ratings. The discrimination parameter and difficulty parameter are fitted through GRM. The discrimination parameter a is the slope of the item characteristic curve (ICC) fitted by GRM, and its value range is usually defined as (0.3, 4). The difficulty parameter b represents the position of the ICC in the capability coordinate, and its value range is normally defined as (−3, 3). Difficulty parameters corresponding to different grades of Likert should gradually increase with each grade.

### 2.7 Exploratory Factor Analysis and Confirmatory Factor Analysis

We determined the final factor structure of CRS‐PCFT through exploratory factor analysis (EFA). Samples come from the pilot survey. The Kaiser–Meyer Olkin value and Bartlett's Sphericity test were used to validate if the data is suitable for EFA. The verification process includes determining the number of common factors extracted through Cattell scree plot evaluation and the structure for each factor. The item will be deleted if the corresponding main factor loading is less than 0.5.

EFA can identify the potential factors of scale, but the specific relationship between factors and observed items requires further investigation. For EFA, the factorloading and crossloading show the explanatory power between items and potential variables. The factorloading is usually higher than 0.5 and crossloading less than 0.4. confirmatory factor analysis (CFA) can accurately assess the explanatory power between factors and items and further clarify the reliability and validity of the scale. The data from 398 formal survey samples was used for CFA. A CFA model was contributed and modified. On this basis, a second‐order CFA model was constructed to test the explanatory power of the extracted factors on the concept of common factors. CFA is usually finished by the structural equation model (SEM). The index of the chi‐square degree of freedom ratio (*χ* ^2^/df), Root Mean Square Error of Approximation (RMSEA), Goodness of Fit Index (GFI), Adjusted Goodness of Fit Index (AGFI), Normed Fit Index (NFI), Relative Fit Index (RFI), Incremental Fit Index (IFI), Tucker–Lewis Index (TLI), and Comparative Fit Index (CFI) are all fitting indexes of the structural equation model. Generally, the SEM is fitting well when *χ* ^2^/df is less than 3, RMSEA is less than 0.08, GFI, AGFI, NFI, RFI, IFI, TLI, and CFI are all higher than 0.9.

### 2.8 Statistical Analyses

The independent‐sample *t*‐test was used to validate differences in the variances and means among items. The Spearman correlation coefficient was used to observe the correlations of interitem and item‐total. We also compared the relation between CRS‐PCFT and COST through the Pearson correlation coefficient. The differential analyses and EFA were conducted using IBM SPSS Statistics 24. The ltm package for R was utilized to fit the GRM. The CFA was accomplished using IBM SPSS Amos 24.

## 3 Results

### 3.1 Sociodemographic Characteristics

We surveyed 220 and 425 caregivers, respectively, during the pilot and formal survey stages; 14 and 27 caregivers refused our survey request due to taking care of their children or resting. Finally, 206 and 398 valid questionnaires were collected, and the effective rates were 93.636% and 93.647%. Sociodemographic characteristics are shown in Table 1.

**TABLE 1:** Sociodemographic characteristics of the patients with pediatric cancer and their caregivers.

Characteristics	Pilot survey		Formal survey	
	No.	%	No.	%
Patients	206	100	398	100
Sex				
Male	125	60.68	225	56.533
Female	81	39.32	173	43.467
Age				
0–2	39	18.932	89	22.362
3–4	47	22.816	86	21.608
5–6	31	15.049	70	17.588
7–8	33	16.019	52	13.065
9–10	25	12.136	42	10.553
11–12	15	7.282	30	7.538
13–14	16	7.767	29	7.286
Cancer type				
Blood and Lymphatic	27	13.107	73	18.342
Blastoma	103	50	159	39.95
Sarcoma	33	16.019	88	22.111
Germinoma	17	8.252	39	9.799
Others	26	12.621	39	9.799
Caregivers				
Sex				
Male	51	24.757	102	25.628
Female	155	75.243	296	74.372
Age				
≤ 20	1	0.485	2	0.503
21–25	5	2.427	13	3.266
26–30	31	15.049	71	17.839
31–35	60	29.126	127	31.91
36–40	66	32.039	116	29.146
41–45	27	13.107	50	12.563
46–50	16	7.767	19	4.774
Education level				
≤ High school	144	69.903	253	63.568
College	58	28.155	134	33.668
> College	4	1.942	11	2.764
Work status				
Employment	67	32.524	166	41.709
Unemployment	139	67.476	232	58.291

### 3.2 Item Selection

Sixty‐two initial items entered the selection directory. After removing the items unrelated to pediatric and deduplication, 29 were entered into a Delphi consultation. 11 items were combined or removed in the first round of consultation. Two items (13, 29) were removed after the second round. The Kendall W for two rounds was 0.113 (*p* < 0.0001) and 0.142 (*p* < 0.0001). The coefficient of variation (CV) was reduced from 0.095–0.245 in the first round to 0.043–0.213 in the second round. According to the evaluation results, all items have an importance score of 0.8 or higher. The items that were kept and the corresponding importance scores are listed in Table 2.

**TABLE 2:** The composition of CRS‐PCFT items before validation.

Item	Content	Importance
Item 1	I am apprehensive that my child's treatment will put an increasing strain on my family's finances	0.898
Item 3	I feel anxious about the potential decrease in income or job loss for myself or my family members due to childcare responsibilities	0.932
Item 7	I am concerned about my family's financial situation following the child's treatment	0.966
Item 8	I am worried that the child's treatment may have to occupy the family's living expenses	0.881
Item 10	The rising or unexpected medical expenses for my child are causing me pain	0.915
Item 11	I have enough savings, income, or assets to pay for my child's treatment expenses	0.881
Item 12	The child's self‐funded medical expenses far exceeded my expectations	0.898
Item 14	After covering the child's medical expenses, there may be challenges in managing household costs, including other children's education, healthcare, and elderly support	0.915
Item 16	Until now, my child's medical expenses are still within my affordability range	0.864
Item 17	The child's illness has had a significant impact on the family's finances	0.847
Item 18	If necessary, consideration will be given to covering treatment expenses by selling assets such as houses and cars	0.932
Item 19	The additional living expenses accrued during the child's medical treatment, such as rent and transportation, imposed a heavy burden	0.864
Item 23	I need to cut back on household expenses, such as food, clothing, and other consumer goods, to support the child's treatment	0.831
Item 24	Due to financial constraints, I may need to postpone, modify, or forgo certain medical tests or treatments recommended by doctors for my child	0.814
Item 26	To cover my child's medical expenses, I had to seek financial assistance from relatives, friends, or charitable organizations	0.898
Item 28	My family members may go to re‐work or get a part‐time job to relieve the pressure of medical expenses	0.881

**TABLE 3:** Difference Test between the Low and High Groups.

Item	Levene test		Mean Equivalence *t*‐test		
	*F*	*p*	*t*	MD	*p*
Item 1	5.376	0.022	−9.99	1.315	< 0.0001
Item 3	20.631	< 0.0001	−9.234	1.407	< 0.0001
Item 7	28.375	< 0.0001	−9.926	1.296	< 0.0001
Item 8	6.322	0.013	−8.972	1.352	< 0.0001
Item 10	36.735	< 0.0001	−11.427	1.685	< 0.0001
Item 11	5.062	0.027	−13.056	1.722	< 0.0001
Item 12	0.061	0.805	−9.175	1.537	< 0.0001
Item 14	11.747	0.001	−12.93	1.759	< 0.0001
Item 16	8.112	0.005	−11.938	1.833	< 0.0001
Item 17	74.416	< 0.0001	−12.290	1.667	< 0.0001
Item 18	96.961	< 0.0001	−11.032	1.741	< 0.0001
Item 19	33.172	< 0.0001	−13.515	1.704	< 0.0001
Item 23	97.664	< 0.0001	−12.191	1.519	< 0.0001
Item 24	2.766	0.099	−9.841	2.037	< 0.0001
Item 26	46.814	< 0.0001	−13.238	2.019	< 0.0001
Item 28	23.656	< 0.0001	−12.451	1.944	< 0.0001

### 3.3 Item Validation

**FIGURE 1:**
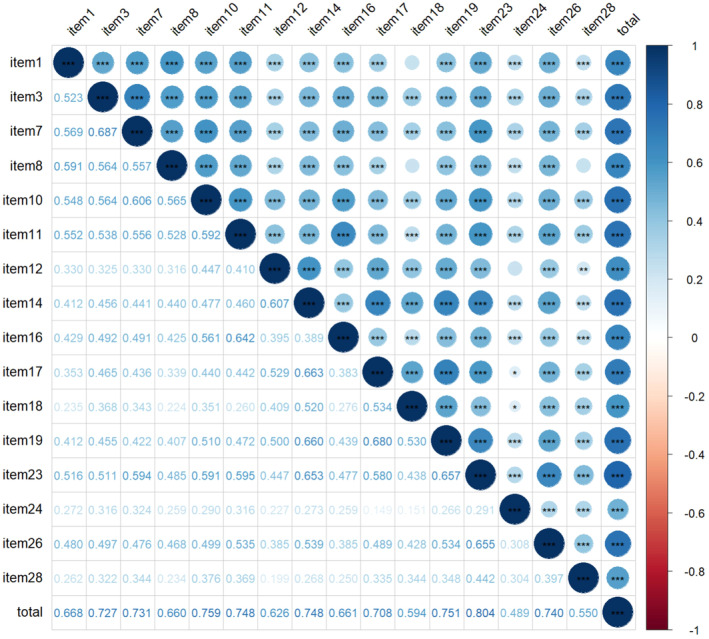
Correlation coefficients among items and total score. The critical ratio method was utilized to compare the difference between the low total scale score group (*N* = 56) and the high group (*N* = 56) among each item. The mean difference (MD) between the low and high groups for each item ranges from 1.315 to 2.037. According to the results of the Levene test and the mean equivalence *t*‐test in Table 3, there is a significant difference in variances and means between the low and high‐score groups for all items (*p* < 0.05) except items 12 and 24. “*” means *p* < 0.05, “**” means *p* < 0.01, “***” means *p* < 0.001.

The scale requires a significant IIC and does not exceed 0.7. Based on the importance score, the lower item will be removed if the IIC of each pair of items is greater than 0.7 or is insignificant. In addition, if the ITC of any item is insignificant or lower than 0.3, it is still necessary to consider removing it from the scale. The correlation coefficients and significance results of IIC and ITC are shown in Figure 1. The circles' size and color depth indicate the correlation's strength. The *p*‐values were marked in the circles. According to the correlation results and the importance score, items 17, 24, and 28 are considered to be removed.

**TABLE 4:** The discrimination parameters, difficulty parameters, and the amount of information of each item.

Item	*a*	*b*				Amount of information	
		*b*_1_	*b*_2_	*b*_3_	*b*_4_	Total	Interval
Item 1	2.291	−2.644	−2.187	−1.156	0.024	6.25	5.54
Item 3	2.074	−2.731	−2.221	−1.183	0.219	5.65	4.88
Item 7	2.081	−3.331	−2.517	−1.38	0.116	6.18	4.7
Item 8	1.777	−2.982	−2.128	−1.062	0.394	4.87	3.96
Item 10	2.316	−2.74	−1.935	−1.053	0.037	6.61	5.78
Item 11	2.225	−3.192	−1.878	−0.856	0.173	6.81	5.43
Item 12	1.508	−2.728	−2.208	−0.917	0.527	3.71	3.06
Item 14	2.445	−2.400	−2.003	−1.075	0.293	6.8	6.33
Item 16	1.733	−3.568	−1.552	−0.469	0.501	5.04	3.72
Item 17	2.623	−2.248	−2.000	−1.233	−0.387	6.41	6.08
Item 18	1.745	−2.163	−1.904	−1.446	−0.394	3.42	3.09
Item 19	2.531	−2.835	−2.006	−1.150	−0.023	7.56	6.55
Item 23	3.507	−2.735	−2.193	−1.286	−0.174	11.37	10.37
Item 24	0.79	−2.111	−0.492	0.275	1.628	1.54	1.07
Item 26	2.536	−2.031	−1.643	−0.894	−0.196	6.13	5.93
Item 28	1.268	−2.467	−1.811	−0.989	0.089	2.58	2.12

**FIGURE 2:**
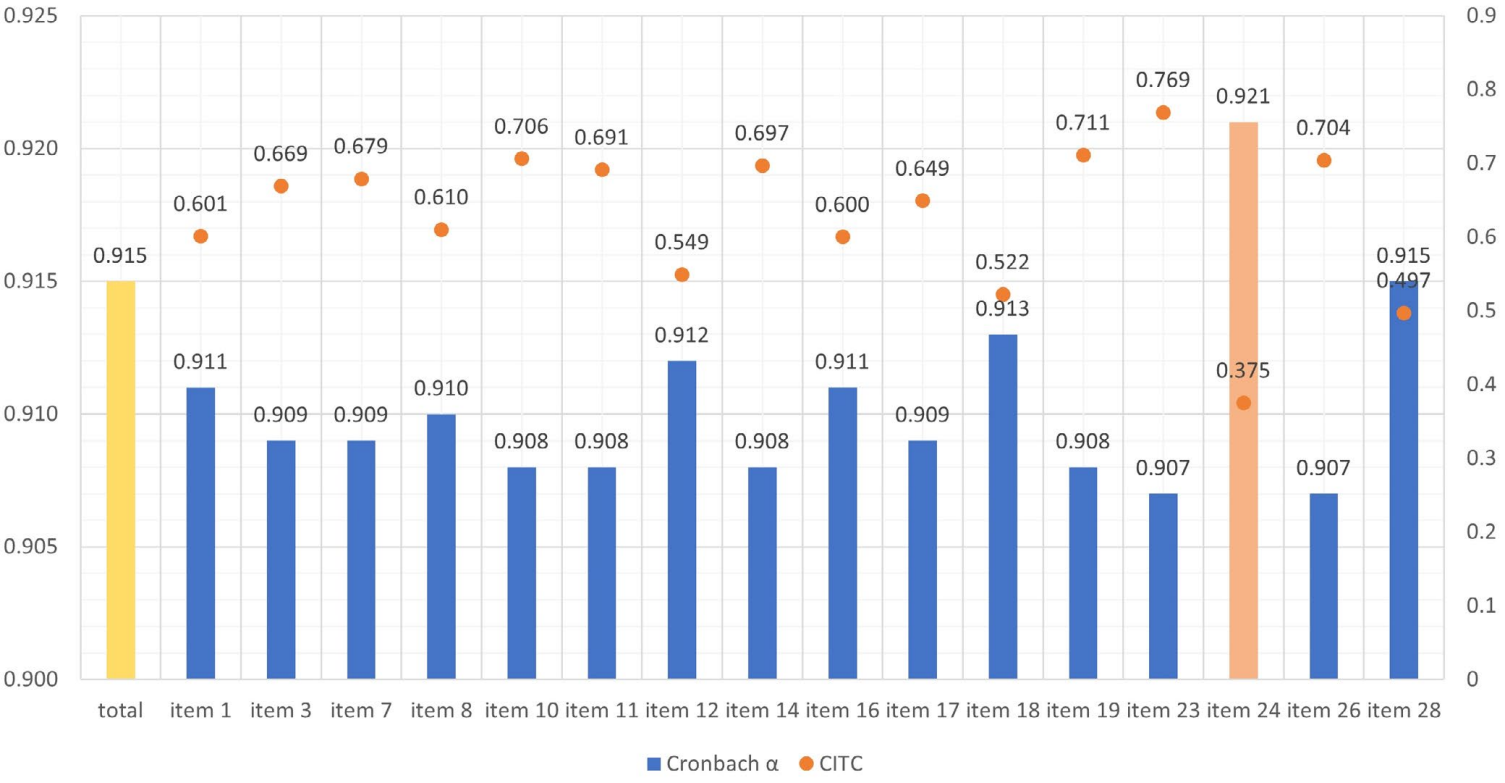
Cronbach α and CITC of Each Item. The application of GRM is based on the assumption of unidimensionality. We have validated the unidimensionality test using principal component analysis, and the results showed that the first initial eigenvalue (7.605) is more than 5 times the second initial eigenvalue (1.487). The hypothesis of unidimensionality holds. According to the model fitting results of GRM shown in Table 4, all items' parameter a are within (0.3, 4). Parameter b of items 7, 11, and 16 are out of (−3, 3). The item characteristic curve (ICC) and item information curve (IIC) of each item are shown in Figure 3. Combining the amount of information, ICC, and IIC for each item, items 7, 11, and 16 should be considered for removal from CRS‐PCFT.

We observed the changes in Cronbach α of CRS‐PCFT after removing each item sequentially and the corresponding CITC. Except for item 24, all others have an ICTC higher than 0.4 and the Cronbach α of CRS‐PCFT had no significant improvement when the item was removed sequentially. The CITC and Cronbach α special changes are shown in Figure 2. Item 24 is considered for removal.

**TABLE 5:** Factor loadings and communalities of each item.

Item	Item contents	Factor loadings		Communality
		1	2	
Item8	I am worried that the child's treatment may have to occupy the family's living expenses.	0.801	0.184	0.675
Item1	I am apprehensive that my child's treatment will put an increasing strain on my family's finances.	0.779	0.176	0.638
Item7	I am concerned about my family's financial situation following the child's treatment.	0.777	0.27	0.677
Item3	I feel anxious about the potential decrease in income or job loss for myself or my family members due to childcare responsibilities.	0.732	0.303	0.628
Item10	The rising or unexpected medical expenses for my child are causing me pain.	0.695	0.368	0.618
Item18	If necessary, consideration will be given to covering treatment expenses by selling assets such as houses and cars.	0.069	0.796	0.638
Item19	The additional living expenses accrued during the child's medical treatment, such as rent and transportation, imposed a heavy burden.	0.304	0.793	0.721
Item14	After covering the child's medical expenses, there may be challenges in managing household costs, including other children's education, healthcare, and elderly support.	0.304	0.782	0.704
Item23	I need to cut back on household expenses, such as food, clothing, and other consumer goods, to support the child's treatment.	0.505	0.673	0.708
Item26	To cover my child's medical expenses, I had to seek financial assistance from relatives, friends, or charitable organizations.	0.474	0.613	0.601
*F* %		34.416	30.668	
Correlation		0.784		

**FIGURE 3:**
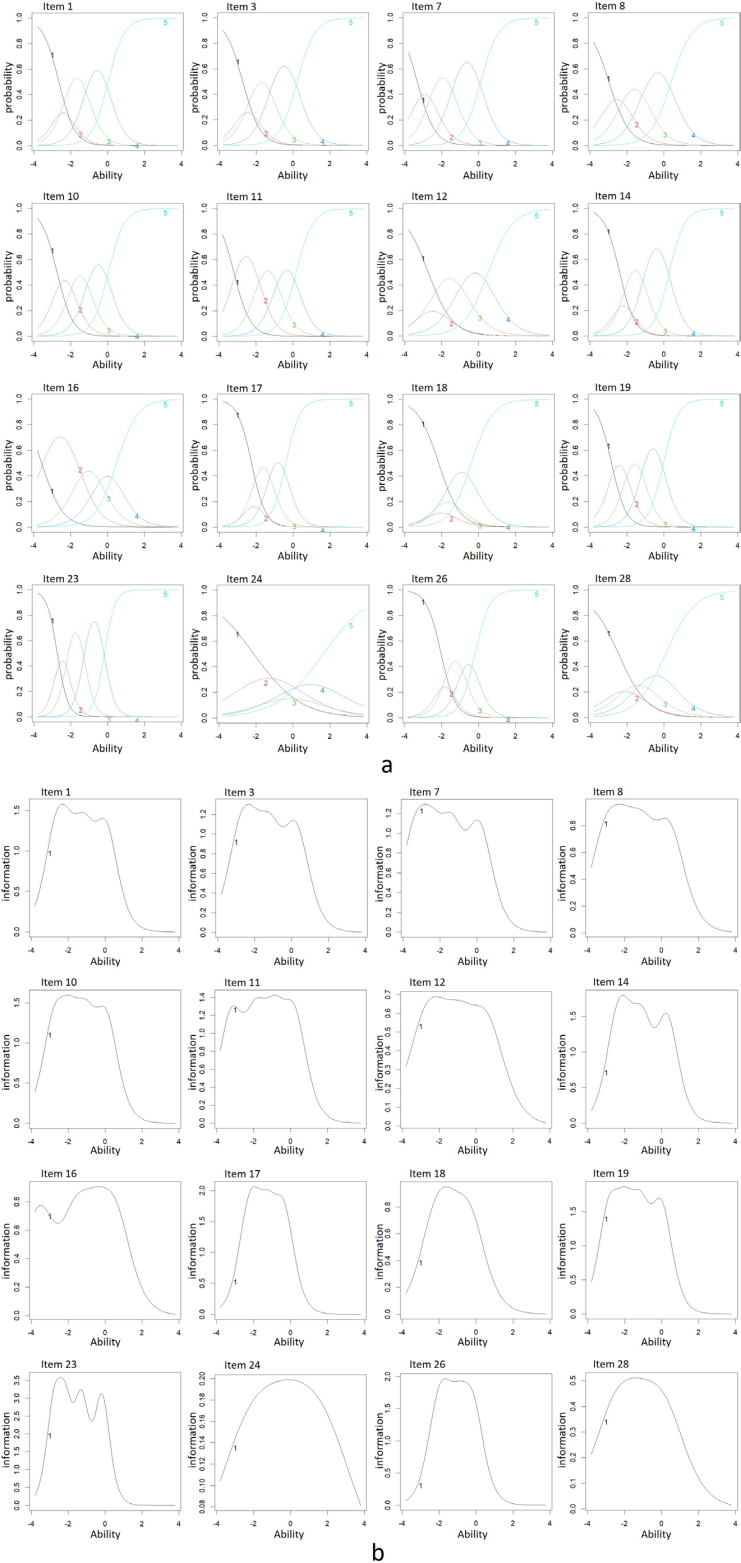
Item characteristic curves (ICC) and item information curves (IIC). A panel discussion confirmed the content of CRS‐PCFT based on the item validation results. 10 items (1, 3, 7, 8, 10, 14, 18, 19, 23, and 26) were retained. The Kaiser–Meyer Olkin value was 0.933 and passed Bartlett's Sphericity Test (*χ* ^2^ = 1737.174, *p* < 0.0001). According to the EFA and the scree plot, two dimensions were extracted: “psychological issues” (PI) and “financial damage and coping behaviors” (FD). The factor loadings and communalities of each item are shown in Table 5. The cross‐loadings for items 23 and 26 are both higher than 0.4. Therefore, we revalidated the structure of EFA by CFA. The chi‐square degree of freedom ratio is 1.865. The root mean square error of approximation (RMSEA) is 0.065 less than 0.08. The structure equation model fitting indexes of the Goodness of Fit Index (GFI = 0.940), Adjusted Goodness of Fit Index (AGFI = 0.902), Normed Fit Index (NFI = 0.942), Relative Fit Index (RFI = 0.923), Incremental Fit Index (IFI = 0.972), Tucker–Lewis Index (TLI = 0.963), and Comparative Fit Index (CFI = 0.972) are all above 0.9. Figure 4 shows that the model fits well, and the scale structure is stable.

**FIGURE 4:**
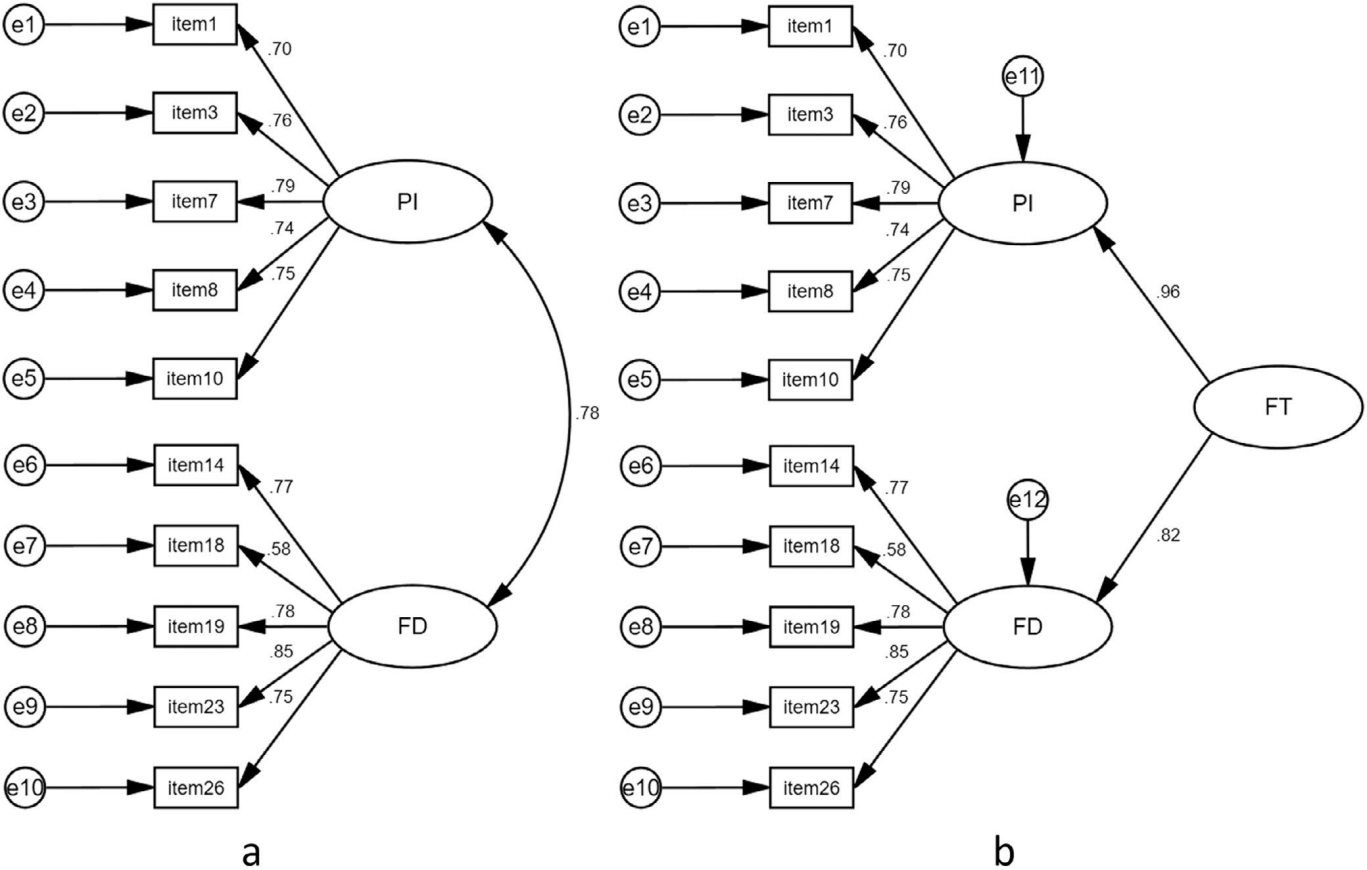
The First and Second Orders of CFA.

### 3.4 Reliability and Validity

The reliability and validity analyses are based on the 398 formal survey participants' data. Cronbach α of CRS‐PCFT is 0.944. The composite reliability (CR) of PI and FD are 0.938 and 0.895 respectively. Research randomly selected 61 caregivers for questionnaire retesting after 1 week, with a correlation of 0.739 (*p* < 0.0001). CRS‐PCFT demonstrates strong reliability and internal consistency.

According to the CFA results, The average variance extracted (AVE) values of factors PI and FD are 0.751 and 0.630, and factor loadings of each item fluctuate between 0.723–0.947. The CRS‐PCFT showed good convergent validity. The correlation coefficient between factor PI and FD is 0.771, which is less than the square root of the AVE values of each factor. CRS‐PCFT demonstrates strong discriminant validity. The comprehensive score for financial toxicity (COST, reverse scoring, a low score means high financial toxicity) was chosen as the criterion scale. Using continuous total scores, the correlation between COST and the two factors of CRS‐PCFT are −0.771 (*p* < 0.0001) and −0.808 (*p* < 0.0001), and −0.843 (*p* < 0.0001) between CRS‐PCFT and COST. Results indicate that CRS‐PCFT can accurately reflect the economic toxicity level of the target population.

### 3.5 CRS‐PCFT Score and Threshold

The score range of CRS‐PCFT is 0–40. The median was 33.5 (mean ± standard deviation, 30.81 ± 8.938). We divided the total scores of CRS‐PCFT into low (less toxicity) and high (more toxicity) groups at the median. Factors included education (*r* = −0.284, *p* < 0.0001), work status (*r* = −0.173, *p* = 0.001), and household income (*r* = −0.253, *p* < 0.0001) are significantly negatively correlated with CRS‐PCFT, and tumor stage (*r* = 0.147, *p* = 0.003) significantly positively correlated with CRS‐PCFT. However, other factors such as cancer type (*p* = 0.399), treatment methods (*p* = 0.426), and medical insurance type (*p* = 0.061) are nonsignificant. Based on Spearman rank analysis, a significant correlation exists between CRS‐PCFT and COST (*r* = 0.469, *p* < 0.0001).

## 4 Discussion

A scale is an essential instrument for accurately assessing the financial toxicity levels within the target population. Keywords such as financial burden [24, 25, 26, 27], financial distress [26, 28, 29, 30], and financial hardship [1, 31, 32, 33] showed the influences of out‐of‐pocket costs on cancer patients or caregivers. However, the effects of economic issues on families dealing with cancer are multifaceted, including their financial situation, psychological well‐being, and coping strategies [8]. The concept of financial toxicity has addressed this issue effectively [4]. Many scholars have developed non‐specific or specific scales to assess the levels of financial toxicity in relevant cancer populations [17, 18, 20, 21]. The COST is currently one of the most highly recognized measurement tools in financial toxicity, and it has demonstrated performance reliability and validity in relevant research [12, 13, 14, 34, 35]. However, a specific financial toxicity measurement scale has not yet been developed for the patients with pediatric cancer and their caregivers. In our survey, the caregivers showed significant reading difficulties and were unaccustomed to COST. It is essential to create a specific scale for this unique population to accurately assess their financial toxicity status.

In the development process of CRS‐PCFT, we referred to widely accepted standards for developing PROMs [36]. We adopted a 5‐level Likert structure similar to COST to measure and evaluate the financial toxicity of the target population [12]. Items of CRS‐PCFT come from other mainstream financial toxicity scales and interview records with childhood cancer caregivers. The Delphi technique ensures the accuracy of each item's expression [37]. We adjusted and removed 13 items from the initial directory through Delphi. The classical test theory (CTT) is commonly used in scale development. Still, it also has certain limitations, such as overly idealized assumptions and an inability to distinguish sources of error [38, 39]. By combining the Item Response Theory (IRT), the rationality of item description and scale structure can be further ensured [39].

Both rounds of surveys meet the sample size requirements for statistical methods [40]. The survey imposes no restrictions on the child's stage of treatment; caregivers can be included in the sample as soon as their treatment begins. This helps ensure the authenticity of the sample distribution. CRS‐PCFT adopted a Likert scale format used in the FACT and COST instruments [12, 41]. 6 items were removed based on the IIC, CR, Cronbach α, and fitting results of GRM. The 10 retained items have made significant contributions to the reliability and validity of the scale. After EFA, two factors (psychological issues, financial damage and coping behaviors) were extracted from 10 items. Altice et al. [42] proposed that the financial burden experienced by cancer survivors comprises three key components: material conditions, psychological responses, and coping behaviors. This aligns with the findings of this study.

The second‐order CFA indicates that two factors can be used to extract a second‐order common factor, referred to as financial toxicity. The validation results from a sample of 398 data points indicate that the scale demonstrates strong reliability and validity. Souza et al. [12, 16] used the median to distinguish between populations with high and low economic toxicity levels on the COST, and only education significantly correlated with financial toxicity levels. The results of CRS‐PCFT showed that lower educational levels and unemployment of caregivers, lower household income, and later stages of the child's tumor are associated with higher levels of financial toxicity. Compared with COST, using the median as a threshold to differentiate between high and low levels of financial toxicity in the target population, CRS‐PCFT has more data support [12]. However, some factors such as cancer type, treatment methods, and medical insurance type still did not show a significant correlation with CRS‐PCFT. There are differences in the availability of resources to families with pediatric cancer patients. In addition, the financial toxicity scores are based on the subjective judgment of respondents. It can be inferred that the financial toxicity level is mainly influenced by the degree of consumption of household economic resources rather than the absolute value of medical expenses. Therefore, it needs to be further validated. In this study, the CRS‐PCFT scores of samples are generally high, which may be linked to the research environment, such as the children's medical insurance system in China, economic conditions, and caregivers' understanding of children's diseases. The threshold needs to be further determined based on additional research. Several pieces of research showed that family financial toxicity is particularly severe in countries with limited resources [43, 44]. Surveys conducted in other countries may yield different results.

There are still some limitations in this research. Due to the characteristics of the hospitals from which the samples were obtained, the disease distribution did not closely align with the pediatric cancer spectrum. However, with the continuous maturity and popularization of high‐end medical technologies such as immunotherapy, proton therapy, and new chemotherapy drugs, the differences in medical costs between different types of tumors are gradually narrowing. The next major task is to verify the applicability of CRS‐PCFT using a pediatric cancer patient sample that better reflects the actual tumor spectrum incidence rate. In addition, the Delphi technique inevitably introduces some degree of subjective bias. Meanwhile, the selection of samples is limited to China, and we will strive to obtain different data from more countries in our next research.

We developed a financial toxicity measurement instrument for the patients with pediatric cancer and their caregivers based on the data from 604 samples collected over two rounds of surveys. The CRS‐PCFT has demonstrated strong stability, reliability, and validity. The patients with pediatric cancer and their caregivers are one of the most economically toxic groups in the cancer population. The CRS‐PCFT lays the groundwork for further research. Verifying the applicability of the CRS‐PCFT, exploring the risk of pediatric cancer financial toxicity, and implementing effective interventions are our next major tasks. We believe that scholars will increasingly focus on the economic challenges faced by this group and validate findings from various perspectives.

## Author Contributions

**Pengfei Li:** methodology, software, data curation, validation, formal analysis, writing – original draft, writing – review and editing, investigation, supervision. **Nan Zhang:** conceptualization, methodology, validation, supervision, project administration, resources. **Xinyue Xu:** methodology, software, data curation, investigation, visualization, resources. **Yan Liu:** conceptualization, methodology, software, investigation, validation. **Zhengyang Lu:** conceptualization, methodology, software, investigation. **Qian Gao:** conceptualization, methodology, software, investigation. **Shihong Lin:** conceptualization, methodology, software, investigation. **Weimin Guan:** investigation, data curation, validation, visualization. **Wenxuan Yan:** conceptualization, methodology, software, investigation, validation. **Boyu Liu:** conceptualization, methodology, software, investigation, validation. **Youhua Lu:** funding acquisition, project administration, resources. **Jinming Yu:** project administration, resources, conceptualization, funding acquisition.

## Conflicts of Interest

The authors declare no conflicts of interest.

## Data Availability

The data that support the findings of this study are available from the corresponding author upon reasonable request.
